# A review on optimization of Wilms tumour management using radiomics

**DOI:** 10.1093/bjro/tzae034

**Published:** 2024-10-08

**Authors:** Maryam Alhashim, Noushin Anan, Mahbubunnabi Tamal, Hibah Altarrah, Sarah Alshaibani, Robin Hill

**Affiliations:** Radiology Department, College of Medicine, Imam Abdulrahman Bin Faisal University, King Faisal Ibn Abd Al Aziz Rd, Dammam 34212, Saudi Arabia; Medical Imaging Services Center, King Fahad Specialist Hospital Dammam, Dammam 32253, Saudi Arabia; Department of Biomedical Imaging, Advanced Medical and Dental Institute, Universiti Sains Malaysia, SAINS@BERTAM, 13200, Kepala Batas, Pulau Pinang, Malaysia; College of Engineering, Imam Abdulrahman Bin Faisal University, Dammam 31451, Saudi Arabia; Oncology center, King Fahad Specialist Hospital Dammam, Dammam 32253, Saudi Arabia; Medical Imaging Services Center, King Fahad Specialist Hospital Dammam, Dammam 32253, Saudi Arabia; Department of Radiation Oncology, Chris O'Brien Lifehouse, Sydney 2050, Australia

**Keywords:** Wilms tumour, paediatric cancers, nephroblastoma, artificial intelligence, machine learning, radiomics

## Abstract

**Background:**

Wilms tumour, a common paediatric cancer, is difficult to treat in low- and middle-income countries due to limited access to imaging. Artificial intelligence (AI) has been introduced for staging, detecting, and classifying tumours, aiding physicians in decision-making. However, challenges include algorithm accuracy, translation into conventional diagnosis, reproducibility, and reliability. As AI technology advances, radiomics, an AI tool, emerges to extract tumour morphology and stage information.

**Objectives:**

This review explores the application of radiomics in Wilms tumour management, including its potential in diagnosis, prognosis, and treatment. Additionally, it discusses the future prospects of AI in this field and potential directions for automation-aided Wilms tumour treatment.

**Methods:**

The review analyses various research studies and articles on the use of radiomics in Wilms tumour management. This includes studies on automated deep learning-based classification, interobserver variability in histopathological analysis, and the application of AI in staging, detecting, and classifying Wilms tumours.

**Results:**

The review finds that radiomics offers several promising applications in Wilms tumour management, including improved diagnosis: it helps in classifying Wilms tumours from other paediatric kidney tumours, prognosis prediction: radiomic features can be used to predict both staging and response to preoperative chemotherapy, Treatment response assessment: Radiomics can be used to monitor the response of Wilms and to predict the feasibility of nephron-sparing surgery.

**Conclusions:**

This review concludes that radiomics has the potential to significantly improve the diagnosis, prognosis, and treatment of Wilms tumours. Despite some challenges, such as the need for further research and validation, AI integration in Wilms tumour management offers promising opportunities for improved patient care.

**Advances in knowledge:**

This review provides a comprehensive overview of the potential applications of radiomics in Wilms tumour management and highlights the significant role AI can play in improving patient outcomes. It contributes to the growing body of knowledge on AI-assisted diagnosis and treatment of paediatric cancers.

## Introduction

Globally, Wilms tumour (WT) is the fourth predominant abdominal cancer among the paediatric patients.^1^ Most of the WT cases are presented between ages 3-5 years. The overall 5-year survival rate of WT patients in the developed countries is 92%. In comparison, the survival rate is 78% in low- and middle-income countries, which can have varying levels of health facilities.^2^ In the field of paediatric oncology, WT constitutes 6% of all childhood cancers.

The treatment of WT is a multimodal approach that includes surgery, chemotherapy, and radiation therapy. The specific treatment plan for each patient will depend on the stage of the tumour, the patient’s age and overall health, and the availability of resources.^3^

It is believed that a WT initiates at the genetic level when abnormal genetic modifications take place during normal embryological development of the genitourinary tract.^4^^,^^5^ Wilms tumour can affect one or both kidneys. More aggressive WT can gradually spread to other body parts, such as the lungs, liver, bone, brain, or nearby lymph nodes. Metanephric tissue or nephrogenic rests are associated with WT development. These genetic abnormality occur in about 1% of children’s kidneys and typically regress during infancy.^6^ All WT are associated with abnormal metanephric cells. However, only about 35% of unilateral WT have been found to have these abnormalities. This suggests that other factors, in addition to metanephric abnormalities, may contribute to the development of WT. Several genetic factors can contribute to the development of WT, including sporadic genetic mutations, only 10% are found to have genetic susceptibility such as Beckwith-Wiedemann syndrome, Denys-Drash syndrome, WAGR (Wilms, aniridia, genitourinary anomalies, and retardation) syndrome.^7^ WTX, WT1, and CTNNB1 genes are altered in 33% of WT.^8^ Other genes related to WT consist of TP53 and MYNC. Sporadic WT is often discovered as an abdominal lump.

At preliminary detection, unilateral WT are mostly unifocal, and only 10% are multifocal whereas 5%-7% are bilateral synchronous tumours.^9^ Bilateral and multifocal WT are more common in children with genetical predisposition. Favourable histology WT with normal nuclei is more prevalent than anaplastic WT with large nuclei. In 25%-30% WT cases, the physical symptoms include fever, abdominal pain, haematuria, anaemia, and hypertension.^10^ While it is unclear whether screening definitively reduces overall mortality or tumour stage, tumours identified through abdominal ultrasound screening are typically smaller than those found during the clinical presentation. This is because surveillance allows for earlier detection.

Ultrasound imaging is the most commonly used imaging modality for the initial evaluation of a suspected WT. It is a noninvasive, readily available, and relatively inexpensive modality that can provide valuable information about the size, and characteristics of the tumour. For a better understanding of the tumour location, CT scan or MRI are performed consequently. In accordance with standard practice, children who have inherent genetic variations that coincide with a >5% chance of WT undergo ultrasound screening every 3 months for up to 7 years of age.^6^

Radiomics is an emerging field of AI that aims to extract quantitative information from medical images to characterize tumours and other diseases. This information can be used to improve diagnosis, treatment planning, and prognosis. The basic idea of radiomics relies on the fundamental principle that tumours exhibit unique phenotypic features that can be reflected in their appearance on medical images. By extracting and analysing these features, radiologists can gain a more comprehensive understanding of the tumour and its potential behaviour. By extracting and analysing these features, radiologists can gain a more comprehensive understanding of the tumour and its potential behaviour.^11^ The radiomics process involves several key steps. Image acquisition involves obtaining medical images using modalities such as CT, MRI, and positron emission tomography (PET). Image segmentation delineates the tumour from surrounding tissue. Feature extraction derives numerous quantitative characteristics from the segmented tumour. Feature selection identifies a subset of these features relevant to the specific task. Finally, feature analysis examines the chosen features to identify patterns and relationships.

Radiomics has developed as a powerful tool in precision oncology, offering promising applications in a variety of tasks, including tumour staging, histological subtypes classification, lymph node metastases characterization, prognosis, and risk assessment.^12^ For example: Pan et al have found that radiomic features extracted from medical images can accurately predict the stage of various cancers, such as lung cancer.^13^ This information is crucial for determining the appropriate treatment plan and prognosis. Moreover, Haider et al, found that radiomics can stratify head and neck squamous cell cancer patients into different risk groups based on their tumour characteristics, allowing for personalized risk assessment and targeted interventions.^14^ Moreover, radiomics may assist clinical decision support by quantifying the underlying biology of the tumour without biopsy or any other surgical invasion.^15^ Despite these advancements, further research is warranted to fully realize the potential of radiomics in WT management. Some areas that require further investigation include standardization of radiomics protocols to ensure reproducibility, validation of radiomics biomarkers in prospective clinical studies, and effective integration of radiomics into clinical decision-making. Additionally, integrating multimodal data, such as genetic, histopathological, and clinical information, with radiomics data could enhance diagnostic accuracy, prognostication, and treatment response prediction. Longitudinal radiomics analysis, examining feature changes over time, may provide valuable insights into tumour progression and treatment efficacy.^3^^,^^15–19^

**Figure 1.**
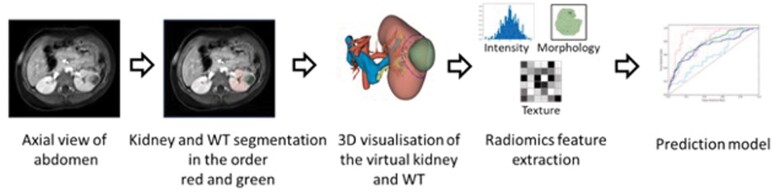
Radiomics workflow for Wilms tumour. The process involves image acquisition, segmentation, feature extraction, selection, and modelling to generate prognostic, diagnostic, or risk assessment predictions. From “Virtual Resection” by van der Zee, J. M., Fitski, M., Simonis, F. F., van de Ven, C. P., Klijn, A. J., Wijnen, M. H., & van der Steeg, A. F. (2022). Virtual resection: a new tool for preparing for nephron-sparing surgery in Wilms tumor patients. *Current Oncology*, 29(2), 777-784.

Figure 1 illustrated the potential of radiomics where radiomics will be applied on the diagnostic images (ultrasound, CT, and MRI) to quantitively address the WT. Decision support system developed using radiomics will in turn improve the present prognosis, diagnosis of WT.^17^ In this review, we provided an overview of conventional imaging methods used in WT management and highlighted the potential of radiomics to improve diagnosis. We also discussed the challenges associated with the application of radiomics in managing WT.

## Clinical practice for WT management

Wilms tumour patients typically show no symptoms during early detection. The condition is often discovered when a paediatrician notices an abdominal lump while examining a child during a routine check-up or when a parent finds an abnormal mass in the child's abdomen while bathing or dressing them. Approximately 35% of patients may experience fever, high blood pressure, blood in the urine, or flank pain.^20^ Occasionally, once a tumour ruptures and bleeds into the nearby tissue, a patient may appear with severe abdominal pain. In the case of clinically suspected WT, physician prescribes a primary abdominal ultrasound. Subsequently, the patient is referred to a paediatric general surgeon and oncologist for successive work-up and treatment. Cancer stage defines tumour size and spreading area from original site of tumour development.^21^^,^^22^

The treatment decisions are based on WT features, histology, and stage.^23–25^ The National Wilms Tumour Study Group (NWTSG) and the International Society of Paediatric Oncology (SIOP) initiated collaborative WT studies to develop protocols. The protocols of NWTSG follows upfront nephrectomy and SIOP protocols follows preoperative therapy. NWTSG protocols focus on precise local staging evaluation, molecular assessment and histologic diagnosis. SIOP protocols focus on chemotherapy for tumour shrinkage to limit tumour rupture risk before nephrectomy. Surgery, chemotherapy and radiation therapy are prescribed by the physician to manage WT of different characteristics and stages. In most cases, the diagnosed child will be treated with combination of surgery, chemotherapy; chemotherapy and radiation therapy or radiation therapy and surgery. Table 1 summarizes the tumour condition is 5 stages of WT.

**Table 1.** Stages of Wilms tumour.

Stage	Description	Figure
Stage I	The tumour is located in the kidney region and no spreading is present. Complete removal with surgery feasible.	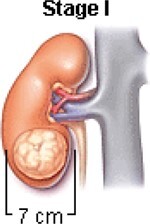
Stage II	Spreading of the tumour beyond the kidney to nearby structures is begins at this stage. Complete removal with surgery feasible.	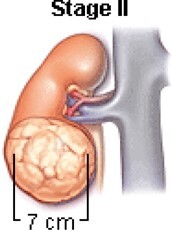
Stage III	Spreading of the tumour beyond the kidney is present. At this stage, tumours spread to lymph glands forming nodes. Complete removal with surgery not feasible.	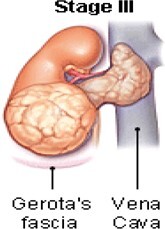
Stage IV	Spreading of the tumour to other parts of the body for example, the lungs, liver. Tumours are at the stage of metastases.	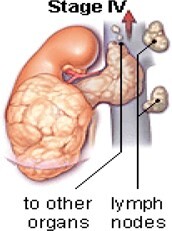
Stage V	Tumours present in both the kidneys also termed as bilateral Wilms’ tumour.	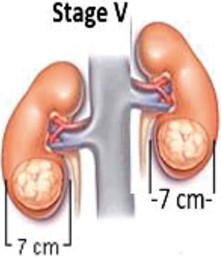

One of the significant development in the field of oncology is the evolution in treatment of WT. The advances in WT treatment was achieved by consistent effort of the NWTSG, the Children’s Oncology Group (COG), and the SIOP, Renal Tumour Study Group (RTSG).^22^^,^^23^ These specialized groups have made remarkable progress, achieving a 90% survival rate and reducing the burden of therapy. A crucial aspect of this advancement has been the development of medical and molecular prognostic indicators, enabling risk-directed treatment. This article reviews the medical and physiological factors used for WT management.

**Figure 2.**
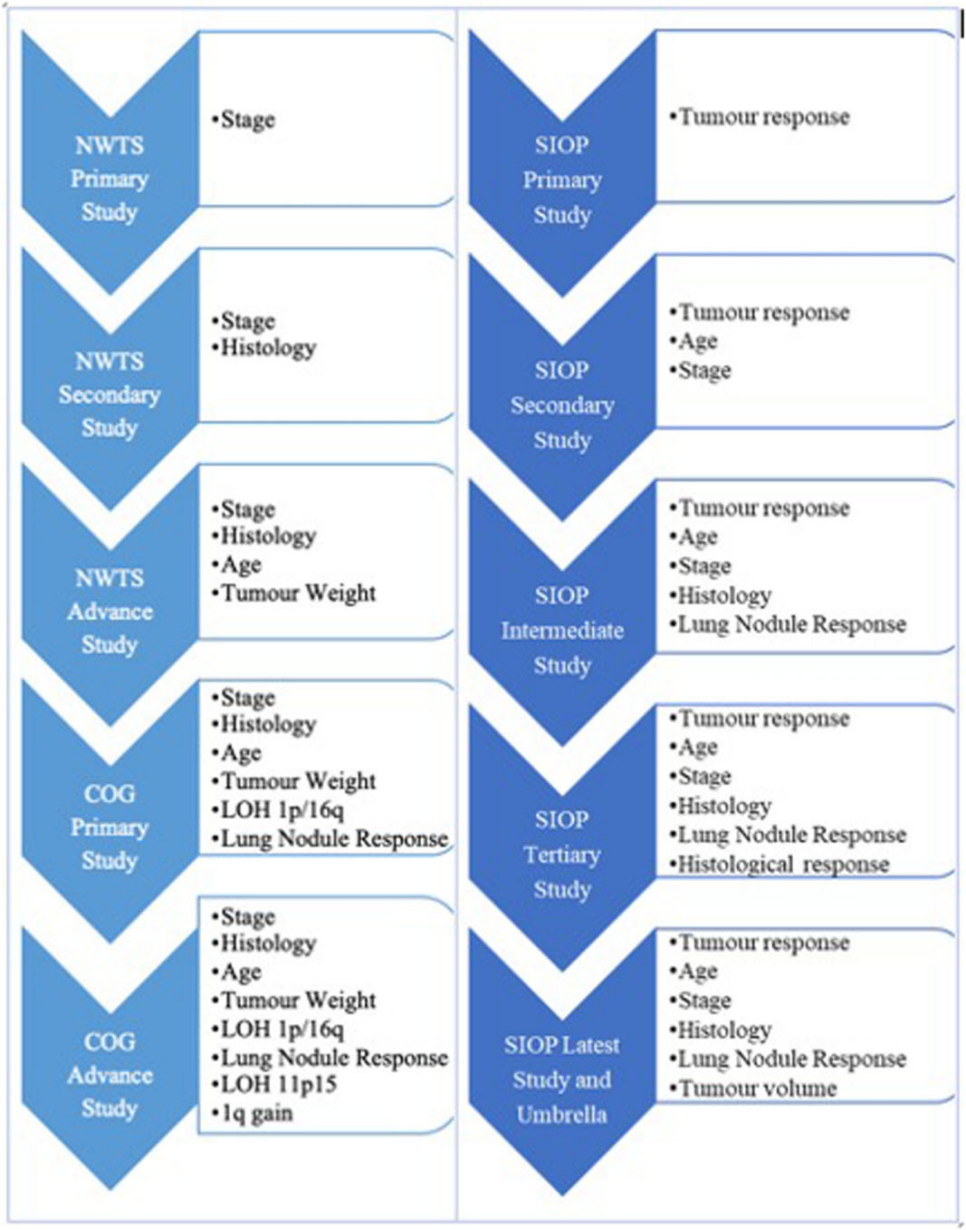
Evaluation of medical and biological factors for Wilms tumour risk classification. This figure summarizes key features assessed in the National Wilms Tumour Study (NWTS)-3, Children’s Oncology Group (COG)-2, and International Society of Paediatric Oncology (SIOP)-5 studies. These factors include tumour stage, histology, tumour weight or volume, patient age, response to therapy, and loss of heterozygosity (LOH) of chromosomes.

Treatment was determined exclusively by tumour stage in the initial NWTSG and SIOP investigations. The risk classification paradigm was expanded throughout the years to include other variables. This expansion supports multidimensional precision medicine strategy. It should be recognized that the associated treatment plan must be taken into account when interpreting prognostic variables. This principle is relevant to WT because COG studies advocate for immediate nephrectomy for most patients whereas SIOP studies advocate for preoperative chemotherapy.^23^  Figure 2 presents an evaluation of medical and biological features used for WT risk classification. As illustrated in Figure 2, in addition to established prognostic factors like tumour stage and histology, clinical trials such as NWTS-5, AREN 0321, AREN 0532, AREN 0533, AREN 0534, AREN 1921, and AREN 2131 have incorporated tumour weight or volume, patient age, response to therapy, and loss of heterozygosity of chromosomes into their risk assessment models.^21^^,^^22^^,^^26–28^

**Figure 3.**
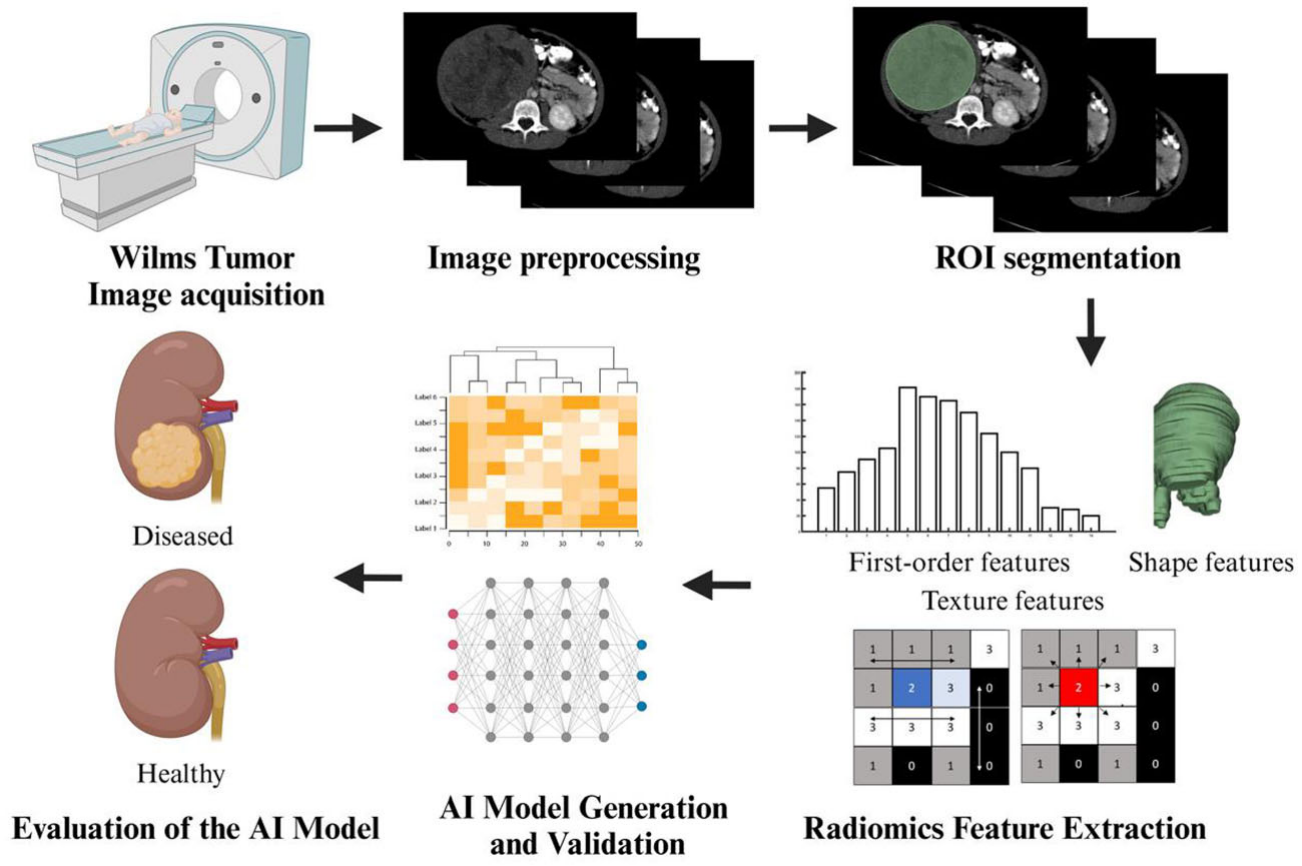
Artificial intelligence (AI)–driven radiomics pipeline for Wilms tumour classification. This figure illustrates the workflow, encompassing image acquisition, segmentation, feature extraction, feature selection, model development, and evaluation. Abbreviation: ROI = region-of-interest.

The early nephrectomy method recommended by the COG enables prompt histological evaluation, molecular examination, and precise local stage assessment. With this information, this knowledge can identify a subset of patients with very low-risk WT who may be treated with nephrectomy alone can be found. Since their beginning in 1971, the SIOP nephroblastoma research and trials have focused on preoperative care. Primary investigation by SIOP demonstrated that neoadjuvant chemotherapy causes favourable stage distribution and inhibits surgical tumour rupture.^23^ The present UMBRELLA regimen resulted from subsequent trials of SIOP that changed postoperative care according to the histology and stage of the tumour.^23^ UMBRELLA protocol concentrates on biomarkers identification and refinement of established biomarkers for the WT management irrespective of tumour type, socio-economic status, or geographic location.^23^ The UMBRELLA protocol aims to establish a data registry encompassing available primary renal tumour information worldwide. The treatment strategy included in the UMBRELLA protocol encompasses guideline for diagnosing bilateral WT, localized and metastatic WT and relapse condition. This guideline is prepared by experts in multiple disciplines including scientists, radiation oncologists, pathologists, radiologists, surgeons, and paediatric oncologists.^23^

The earliest documented prognostic indicator for WT was tumour stage. Advanced tumour stage relayed worse condition of the tumour.^22^ Imaging scans are used to categorize patients’ diagnoses into local (stage I-III), metastatic (stage IV), or bilateral (stage V) disease in both the COG and SIOP protocols. Stage V refers to bilateral disease, and is usually attributed to the worst favourable outcome. Prognostic variations among the same stage group have been discovered by recent research.^26^ The diverse characteristics (positive lymph nodes, positive surgical margins, peritoneal implants, tumour rupture) that define stage III were revealed through a retrospective examination of NWTS to have varying and frequently cumulative effects on the chance of recurrence.^29^

The COG trial AREN0532 investigated a cohort of patients with very small possibility of favourable histology WT. This trial aimed to maximize overall survival with minimum exposure to toxicity. In the COG trial AREN0532, patients with stage III conditions had a 4-year event-free survival rate of 82% with positive lymph nodes as opposed to 94% with negative lymph nodes (*P* < 0.01).^27^ A thorough explanation of lung lumps and their assessment are made prospectively in order to establish a metastatic condition using chest CT scan. The UMBRELLA protocol requires evaluation by standard radiology since standardization alongside the enhancement in imaging investigations is of the foremost significance. The local stage is redefined after preoperative chemotherapy and surgery, determining the degree of postoperative therapy. The span of postoperative therapy ranges from 6 months and more of comprehensive chemotherapy to no further treatment in completely necrotic stage I tumours.^23^

## Imaging of WT

Imaging of nephroblastoma has generally been of a qualitative nature, used for staging and to determine image-defined risk factors. Imaging has been used to describe features such as tumour location, invasion, or compression of structures such as the spinal cord or blood vessels, necrosis, calcification, haemorrhage, and other technique or modality-related characteristics. Apart from tumour dimensions, quantitative features have been less commonly reported in radiology reports.

The first-line technique for assessing paediatric kidney tumours is ultrasonography (US). Ultrasonography helps in localizing the mass to the kidney and separating it from other renal mass causes, such as hydronephrosis. Radiation-free imaging, simplicity of performance, brief examination period is exclusion of sedation makes ultrasound a preferred choice for physical abnormality evaluation in children. On ultrasonography, the WT appears as a solid mass that might have capillary permeability and smooth borders. Cystic or necrotic areas can seem anechoic or hypoechoic, whereas calcification, fat body, haemorrhage can appear as hyperechoic. However, proper outlining of WT using ultrasound exclusively is a very difficult to perform.

CT and MRI allow for the advanced characterisation of tumours by measuring diffusion limitation, attenuation, and enhancement patterns. However, it is still difficult to distinguish malignant kidney tumours in children with imaging. Additionally, MRI costs more, takes longer examination periods, and requires anaesthesia for children while CT poses radiation risks. Therefore, it is important to identify innovative imaging features from the primary modality, US, for discriminating paediatric renal tumours.

In the past 20 years, numerous advanced imaging methods have emerged, including CT/MRI-based radiomics, contrast-enhanced ultrasonography, dynamic contrast-enhanced imaging, dual-energy CT, intra-voxel incoherent motion (an alternative diffusion-weighted imaging [DWI] model), MRI-based DWI, arterial spin labelling, and blood oxygenation level-dependent imaging.^30^ Diffusion-weighted imaging, one of these techniques, is an MRI sequence used to detect restricted diffusion and is now routinely employed in healthcare to aid in lesion diagnosis. However, DWI has limitations, as it may not reliably differentiate between nephroblastoma and WT or determine the histological grade of renal tumours.^31–33^ The other methods have currently been used to paediatric renal tumours, and they have been studied for the characterization and subtyping of adult renal tumours.

## Application of radiomics in WT management

Radiomics can be defined as a quantitative approach to medical imaging, which aims at enhancing the existing data available to clinicians through advanced mathematical analysis.^34^ In other words, radiomics is the evaluation and exploration of quantifiable image-based features from medical images, such as CT or MRI, to describe phenotypic characteristics (ie, signal intensity or contrast enhancement) of a tumour.^35^  Figure 3 illustrates a simplified radiomics pipeline for Wilms tumour classification into disease and healthy. The role of radiomics has been increasing over the past decade, not only for diagnostics but also for prognostics and the ability to predict clinical outcomes in cancers.^36^ In some conditions, such as prostate and breast cancer, quantitative radiomic features using machine learning have been derived that are highly predictive of outcomes.^15^^,^^37^ However, corresponding radiomic features have not yet been defined for nephroblastoma. The heterogeneous feature present in WT is due to the presence of necrosis or haemorrhage, and calcification in 15% of patients.^38^ The discovery of the imaging signatures would be very useful in this heterogeneous cancer, not only for better risk stratification but also to predict outcomes and guide management decisions.

With the increasing number of pattern recognition tools and larger studies, models are being developed to enhance diagnostic, prognostic, and predictive accuracy. In the paradigm of precision medicine, this allows for better differentiation between high-risk and non-high-risk neuroblastoma patients. Neuroblastoma, a cancer that originates in nerve cells, often in the adrenal glands, may invade the kidney, making it crucial to distinguish it from nephroblastoma, a type of kidney cancer. Accurately identifying high-risk neuroblastoma patients, who have <50% survival, ensures they receive more aggressive treatment, while non-high-risk patients, with over 90% survival, can be treated less aggressively.^39^

Globally, COG and SIOP protocols are employed for WT diagnosis.^6^ SIOP concentrates on reducing tumour burden before deciding on any surgical procedure and COG follows resection followed by treatment. Radiomics analysis holds the potential of noninvasively assessing tumour burden. The application of radiomics can reduce surgical intervention thus achieving minimally invasive treatment. Present diagnosis of WT relies on visual perception of the tumour. Radiomics can assist the visual perception by providing quantitative information encoded in the diagnostic image. The studies associated with the application of radiomics for WT management are presented in Table 2. Shin et al concentrated on classifying paediatric tumours utilizing radiomics features.^40^ This study included 32 children. Among them 24 had WT, 5 had cell sarcoma and 3 had rhabdoid tumours. This study is significant because they employed grey-scale ultrasound images to distinguish between textural analysis and paediatric malignant kidney tumours. They reported several second-order statistics features that identified WT from clear cell sarcoma and rhabdoid tumour. These features were also found to have promising diagnostic performances with area under the curve (AUC) values higher than 0.89. For diagnostic identification, the reported technique may be more useful than employing nontexture-based tumour characteristics. However, the study did not examine the intra- and interobserver variabilities present in the image segmentation. The presence of asymmetric distribution of patients in each tumour was overlooked because of the natural incidence of WT, cell sarcoma, and rhabdoid tumours. As a result, no significant features were found to differentiate clear cell sarcomas and rhabdoid tumours.

**Table 2.** Overview of the literature on application of radiomics, machine learning and deep learning for Wilms tumour diagnosis.

No	Study objective	Study population	No of participants	Mean age (months)	Imaging modality	Image collection period	Study type	Radiomics	Deep learning	Machine learning	Ref.
1	Development of machine learning model for predicting preoperative chemotherapy response in Wilms tumours	Preoperative	63	48	CT		Retrospective	No	No	Yes	41
2	Development of automated segmentation technique	Preoperative	45	39	MRI	2018-2020	Retrospective	No	Yes	No	3
3	Deep learning model for differentiating undiagnosed Wilms tumour from other paediatric renal tumours	Preoperative	364	38	CT	2008-2020	Retrospective	No	Yes	No	44
4	Establish radiomic nomogram for nephron-sparing surgery feasibility prediction	Preoperative	58	13	CT	2008-2019	Retrospective	No	No	Yes	16
5	Differentiation of Wilms tumour from clear cell sarcoma and rhabdoid tumour	Preoperative	32	31	Ultrasound	2002-2017	Retrospective	Yes	No	No	5, 40
6	Development of machine learning model for identification of Wilms tumour stage	Preoperative	118	25	CT	2014-2021	Retrospective	Yes	No	Yes	17
7	Development of machine learning model for Wilms tumour prediction without renal capsule, sinus vessel, and lymph node involvement	Preoperative	105	25	CT	2013-2020	Retrospective	Yes	No	Yes	18

A study focused on building a new computer-based prediction model to assess the response of preoperative chemotherapy in WT.^41^ The system was reported to attain 95% overall accuracy. They adopted dataset encompassed 64 patients. The technique consisted of 6 steps starting with region-of-interest (ROI) segmentation and ending with the development of the prediction model. Wilms tumours was characterized before chemotherapy by texture, shape, and functionality-based feature extraction. However, the dataset was small and automated and semi-automated segmentation techniques were not evaluated.

A research led by Ma et al focused on developing machine learning model for preoperative WT stage prediction.^17^ The study population was 118 patients and in total, 1781 imaging-based features were extracted from the ROI of each tumour from the contrast-enhanced CT images. 79% accuracy was reported for the support vector machine model (SVM).

Post-chemotherapy [^18^F]fluorodeoxyglucose-PET was used to evaluate the response of WT after preoperative chemotherapy in another study.^42^ The reduction in tumour size was assessed using an MRI scan of the initial tumour site. Additionally, a research group validated a CT-based nomogram through imaging analysis to preoperatively predict the grades of clear cell renal cell carcinoma. Their study, which included radiomics features extracted from CT images of 258 patients, demonstrated that their developed radiomics signature, consisting of 20 features, performed very well in distinguishing nuclear grades in both the training (AUC = 0.929) and validation (AUC = 0.876) groups.

A study performed by Buser et al. reported that deep learning outperforms manual segmentation of WT.^3^ In the conventional volume assessment, tumour volume is often underestimated and this can be resolved by the application of deep learning in MRI imaging. However, the study incorporated only 6 bilateral WT in their dataset. Hence, their findings need to be validated using a large dataset. Another limitation of this study was that the MRI images were post chemo. Study on diagnostic MRI is also required.

Another study led by Zhou et al demonstrated the importance of AI in differentiating WT from non-WT using CT images.^43^ Their study included 364 children, of whom 269 were WT patients. The developed method accurately distinguished WT from non-WT, achieving accuracy comparable to that of radiologists with 15 years of experience. Manual delineation of the ROI was performed before generating the model. MRI images were not included in this study, as the use of MRI for WT management has not yet gained widespread acceptance.

Zhenwu Li et al focused on developing a prediction model assessing the feasibility of NSS in bilateral WT patients.^16^ Previous literature has already elaborated on the importance of developing a scoring system for NSS feasibility evaluation. This study developed a radiomics nomogram for NSS feasibility evaluation utilizing adult renal tumour scoring system. Their developed nomogram may assist physicians in making NSS decisions. However, the study had several limitations. Selection bias was present in the collected information and the sample size is limited due to low number of incidence of bilateral WT. External validation of their reporting is also recommended as the study was validation was performed internally only.

A study focused on predicting stages of WT utilizing the combination of CT radiomics and machine learning.^17^ The SVM machine learning model had 15 features and successfully performed WT staging with 0.79 accuracy. The results of this research may help the SIOP system design more individualized preoperative chemotherapy regimens. Patients identified as stage I may have the prospect of receiving lower chemotherapy so that treatment-related toxicity can be deducted. Low dose also lowers the risk of late side effects and enhances efficacy as there are significant differences in metastasis and tumour thrombosis between the stage I and non-stage I groups. However, this radiomics investigated the mass of WT only and excluded lymph nodes. This assumption limits the predictive power of the developed radiomics model.

Xiao et al reported the radiomic features of preoperative nephroblastoma as independent predictor of localized nephroblastoma.^18^ The LR-based radiomic model noninvasively predicted nephroblastoma without lymph node, renal capsule and sinus vessels involvement. The reported features may assist surgical decisions system for precise treatment and improve the long-term quality of life of nephroblastoma patients. Investigation of clinical features and genomics was out of scope in this study. Combining data from many dimensions may help the model’s predictive power even more, improving clinical decision system.

## Challenges

Despite the growing applications of radiomics, significant methodological and clinical challenges persist. Extracting a vast number of features from images poses a risk of overfitting and inaccurate predictions. Selecting the most predictive features while maintaining clinical relevance is complex and requires advanced statistical and machine learning techniques. Additionally, establishing inter- and intra-observer reproducibility, selecting appropriate software, and mitigating bias throughout the process are time-consuming challenges. Acquiring large, high-quality datasets is essential for developing clinically relevant models, but this process is also resource-intensive. To achieve routine clinical adoption, research must focus on reducing bias, standardizing protocols, and automating processes to create reliable and accurate radiomics models.

## Conclusion

In paediatric oncology, WT occupy an essential part. Radiomics, machine learning, and deep learning techniques may be utilized to establish the confirmed presence of WT rather than other less frequent renal masses such as clear cell renal sarcoma, rhabdoid tumour, and renal cell carcinoma. Different neoadjuvant chemotherapy regimens could be used to treat these tumours according to their differences for more individualized care. These methods could also be utilized to carry out radio-genomic research on such children. It is possible that radiomic or deep characteristics can be utilized for determining histological categorization in the context of kidney tumours without a biopsy. These methods can assist in distinguishing nephroblastoma from other kidney tumours in children with nephroroblastic tumours. Radiomics and deep learning can reveal instances of clinically significant genetic changes, including the amplified N-myc oncogene. It is conceivable that AI algorithms may be able to give insightful data at the stage of diagnosis, such as the prognosis of treatment response or overall survival. In addition to recognizing symptoms like tumour rupture or metastatic disease which indicate the need for adjustments in oncologic care, the radiologist is crucial in assisting with decisions on surgical interventions. The radiologist can also guide surveillance imaging after therapy has been completed in a way that balances the risk factors of radiation exposure with the ability to detect recurrence. The possibility to personalize treatment for WT has never been better because of the application of novel clinical and molecular prognostic variables. Recent clinical studies have shown that improved therapy can mitigate the effects of several unfavourable prognostic variables. The ongoing SIOP UMBRELLA protocol and future COG trials seek to further refine prognostic factors to enhance precision medicine for WT. Future COG trials and the current SIOP UMBRELLA regimen both aim to improve WT precision medicine by further refining prognostic variables. This review may help researchers to improve and advance radiomics therapy for WT management.
