# Correction to: A review on optimization of Wilms tumour management using radiomics

**DOI:** 10.1093/bjro/tzae044

**Published:** 2024-12-11

This is a correction to: Maryam Alhashim, Noushin Anan, Mahbubunnabi Tamal, Hibah Altarrah, Sarah Alshaibani, Robin Hill, A review on optimization of Wilms tumour management using radiomics, *BJR|Open*, Volume 6, Issue 1, January 2024, tzae034, https://doi.org/10.1093/bjro/tzae034

In the originally published version of the manuscript, the caption to Figure 1, a necessary citation was erroneously omitted. This has now been included and the paper corrected. The caption to this figure now reads: **“Figure 1. **Radiomics workflow for Wilms tumour. The process involves image acquisition, segmentation, feature extraction, selection, and modelling to generate prognostic, diagnostic, or risk assessment predictions. From “Virtual Resection” by van der Zee, J. M., Fitski, M., Simonis, F. F., van de Ven, C. P., Klijn, A. J., Wijnen, M. H., & van der Steeg, A. F. (2022). Virtual resection: a new tool for preparing for nephron-sparing surgery in Wilms tumor patients. Current Oncology, 29(2), 777-784.” instead of: “**Figure 1.  **Radiomics workflow for Wilms tumour. The process involves image acquisition, segmentation, feature extraction, selection, and modelling to generate prognostic, diagnostic, or risk assessment predictions.”

